# A Mathematical Model of Idiopathic Pulmonary Fibrosis

**DOI:** 10.1371/journal.pone.0135097

**Published:** 2015-09-08

**Authors:** Wenrui Hao, Clay Marsh, Avner Friedman

**Affiliations:** 1 Mathematical Biosciences Institute, The Ohio State University, Columbus, OH, United States of America; 2 Health Sciences Center South, West Virginia University, Morgantown, WV, United States of America; 3 Mathematical Biosciences Institute & Department of Mathematics, The Ohio State University, Columbus, OH, United States of America; univeristy of alabama at birmingham, UNITED STATES

## Abstract

Idiopathic pulmonary fibrosis (IPF) is a disease of unknown etiology, and life expectancy of 3-5 years after diagnosis. The incidence rate in the United States is estimated as high as 15 per 100,000 persons per year. The disease is characterized by repeated injury to the alveolar epithelium, resulting in inflammation and deregulated repair, leading to scarring of the lung tissue, resulting in progressive dyspnea and hypoxemia. The disease has no cure, although new drugs are in clinical trials and two agents have been approved for use by the FDA. In the present paper we develop a mathematical model based on the interactions among cells and proteins that are involved in the progression of the disease. The model simulations are shown to be in agreement with available lung tissue data of human patients. The model can be used to explore the efficacy of potential drugs.

## Introduction

Idiopathic pulmonary fibrosis (IPF) is a disease in which scar tissue in the lung is deposited; the deposition of the scar tissue is called fibrosis. As the disease progresses, alveolar-capillary units are impacted, oxygen and carbon dioxide exchange is impaired, ultimately leading to respiratory failure. IPF usually affects older people [1], but its etiology is unknown. IPF has no cure yet, and life expectancy is 3-5 years after diagnosis [2]. IPF is characterized by repeated injury to alveolar epithelium. The injury results in loss of alveolar epithelial cells (AECs) due to increased apoptosis, epithelial to mesenchymal transition (EMT), and abnormal tissue repair [3]. Oxidative stress is associated with the disregulation of the AECs [4, 5], and inflammation is initiated by damaged AECs [6]. Fibrocytes, bone marrow mesenchymal progenitor cells circulating in the blood, play a role in wound repair and are increased in lungs of patients with IPF. However, fibrocyte numbers do not correlate with disease severity [7, 8].

Inflammation and injury activate AECs [9, 10, 11], and activated AECs secrete a number of pro-inflammatory mediators including tumor necrotic factor alpha (TNF-*α*) [12, 13] and chemoattractant monocyte chemotactic protein-1 (MCP-1) [7, 14, 15]. MCP-1 recruits circulating monocytes from the blood into damaged lung tissue, where they differentiate into classically activated macrophages M1.In normal lung tissue (homeostasis), macrophages from blood monocytes develop into alveolar macrophages (AM) [12, 16]. Alveolar macrophages are often referred to as alternatively activated macrophages, or M2 macrophages. However M2 macrophages are heterogeneous, and in IPF there appears to be a shift from monocyte-derived M1 macrophages to pro-fibrotic M2 macrophages [17, 18]. These M2 macrophages are responsible for the progression from inflammation to interstitial fibrosis [2, 18] by secreting platelet-derived growth factor (PDGF) [19, 17], transforming growth factor-beta (TGF-*β*) [17], matrix metalloproteinase (MMP) and tissue inhibitor of metalloproteinase (TIMP) [17], all of which are involved in the regulation of tissue fibrosis. TGF-*β* is produced also by fibroblasts activated by AEC [12, 20]. Both TGF-*β* and reactive oxygen species increase AEC apoptosis [20].

TNF-*α* is produced by the proinflammatory macrophages as well as by activated AEC, and it induces polarization of M2 into M1 [21] which helps to resolve the fibrosis. This polarization by TNF-*α* is resisted by IL-13 [22, 23, 24] which is produced by M2 macrophages and TH2 lymphocytes [25]. On the other hand, MMP28 [26] and other extracellular matrix (ECM) molecules (e.g. monomeric collagen type 1 interacting with CD204 on M1 [17]) activate polarization of M1 into M2 macrophages. TGF-*β*, along with AEC-derived basic fibroblast growth factor (bFGF) increase the proliferation of interstitial fibroblasts [6, 20]. PDGF and TGF-*β* transform fibroblasts into myofibroblasts [27, 28, 29, 30], which together with fibroblasts produce ECM. Imbalance between MMP and its inhibitor TIMP facilitates the accumulation of ECM and the formation of fibrosis [31].

**Fig 1:**
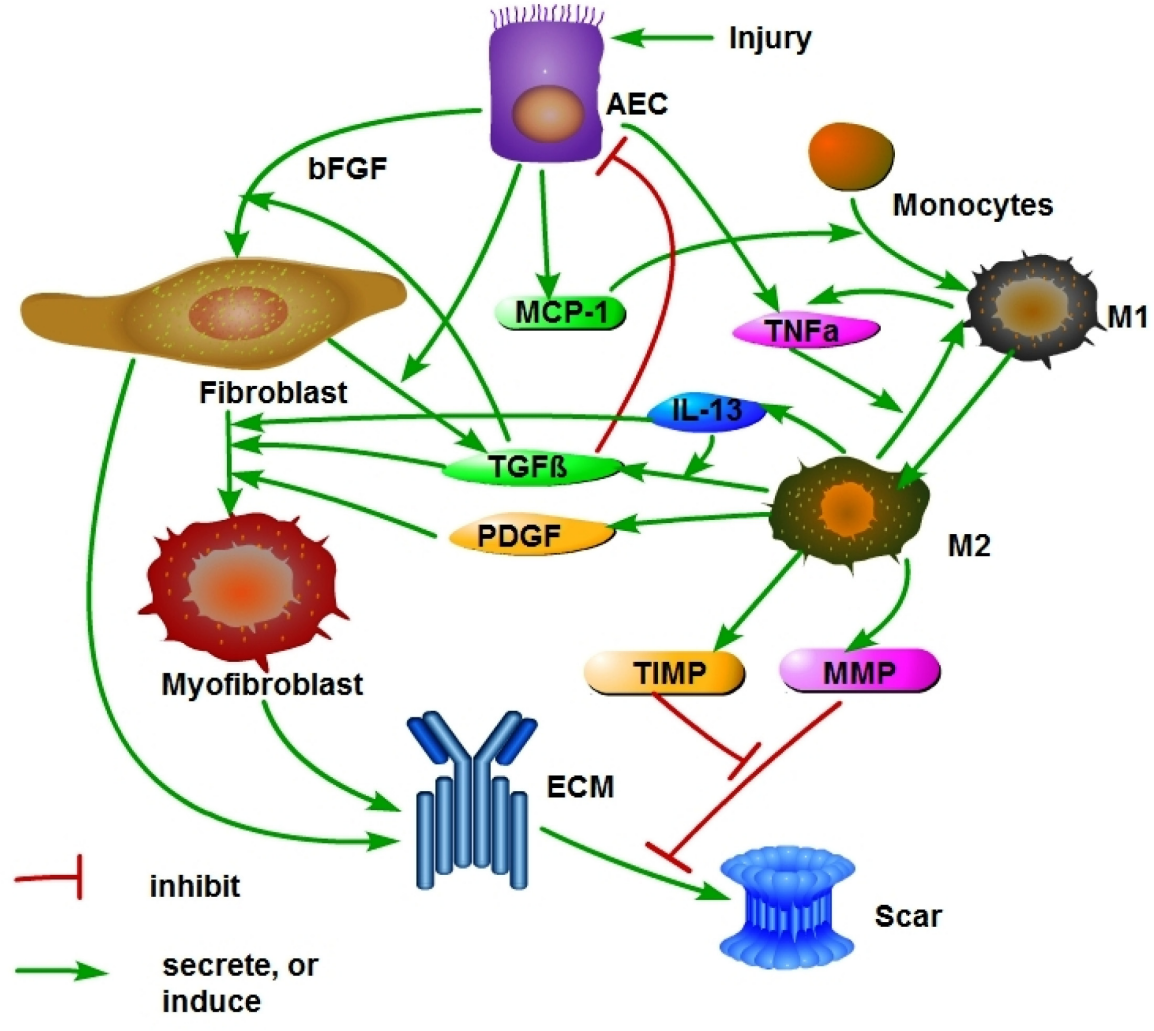
Schematic network of cells and proteins in IPF.

Fibrosis is a disease in which scar tissue develops in an organ resulting in loss of functionality of the organ. Although this process evolves in nearly identical way in all organs, there may be some aspects which are organ specific. Recently Hao et. al. [32] developed a mathematical model of renal interstitial fibrosis and demonstrated that the model can be used to monitor the effect of treatment by anti-fibrotic drugs that are currently being used, or undergoing clinical trials, in non-renal fibrosis. The present paper is based on the model developed in [32] but in addition in includes two features that are unique to pulmonary fibrosis. The first one is the fact that in lung fibrosis we need to deal with two phenotypes of macrophages: monocyte-derived inflammatory macrophages (M1) and anti-inflammatory alveolar macrophages (M2). The network shown in Fig 1 is similar to the network in Fig 1 of [32], but in the present figure the macrophages are divided into M1 and M2 phenotypes, and they play different roles in the fibrotic process.

**Fig 2:**
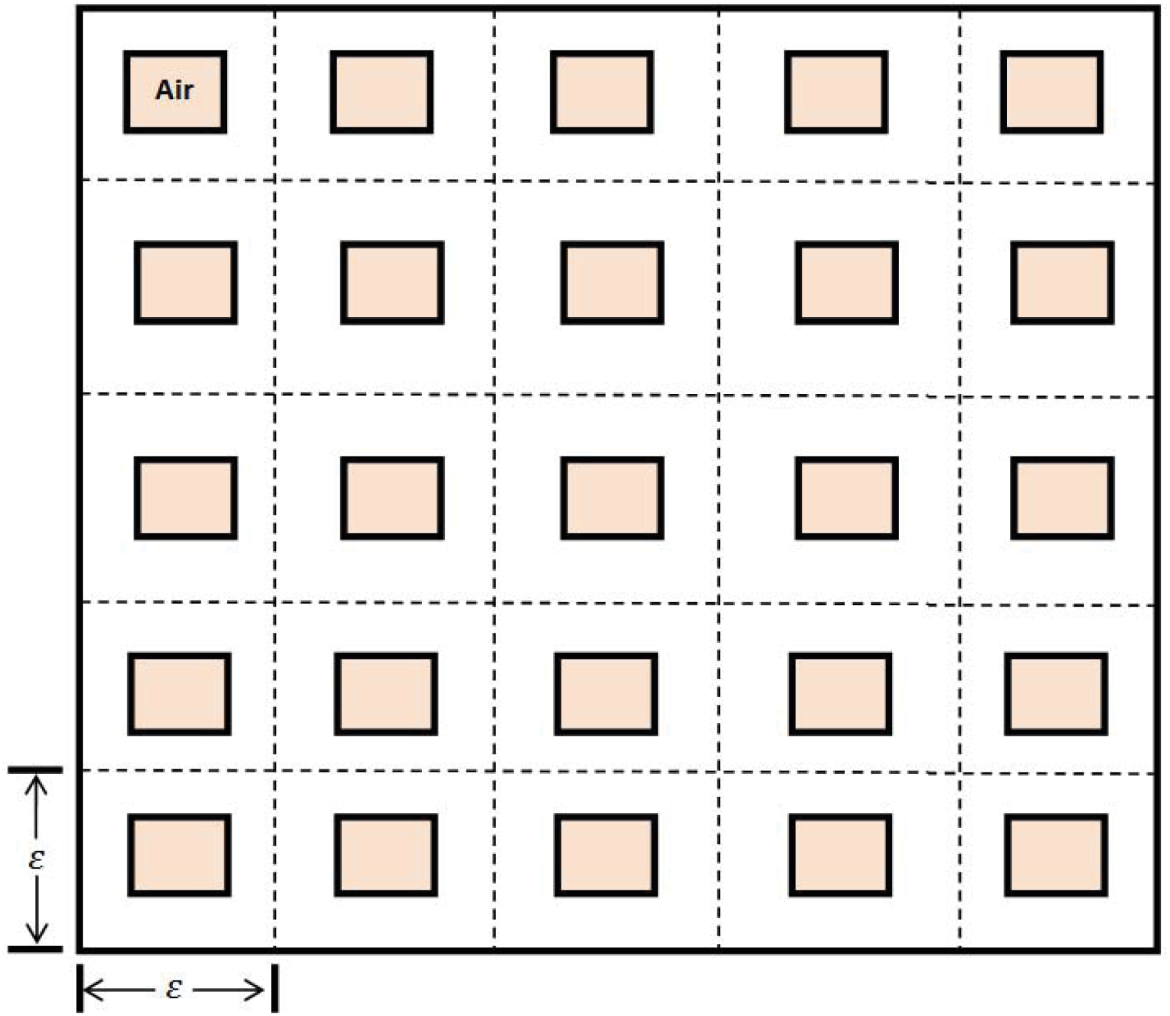
Lung geometry consists of a periodically arranged cubes with smaller cubes representing the air space of alveoli.

The second unique feature in lung fibrosis is the geometry of the lung which includes a very large number of alveoli. This complex geometry is represented, in a simplified form, in Fig 2. Our mathematical model of IPF is based on Fig 1 combined with ‘homogenization’ method associated with Fig 2.

The present paper develops for the first time a mathematical model of IPF. The model is based on the experimental and clinical information referenced above, schematically summarized in the network shown in Fig 1. The model is represented by a system of partial differential equations. The model is validated by comparing the simulation results with patients data and may be used to test the efficacy of potential drugs in stopping the patient’s growth of fibrosis.

## Materials and Methods

### Mathematical model

Table 1 lists all the variables of the model in units of *g*/*cm* ^3^. For the purpose of mathematical modeling we use a simple representation of the lung geometry, whose 2-dimensional projection is shown in Fig 2. The tissue under consideration is a cube R with edge-size 1 cm. The cube is partitioned by periodically arranged small cubes *T*_*ɛ*_ with edge-size *ɛ*, and in each *ɛ*-cube there is a concentric cube *A*_*ɛ*_ of edge-size (1 − *θ*)*ɛ*; the *A*_*ɛ*_ represent the alveoli air space, and the domains *T*_*ɛ*_/*A*_*ɛ*_ represent the alveolar tissue. An alveolar diameter is approximately 140 *μ*m [33] and the thickness of the arterial wall which contains the capillaries, epithelial cells and fibroblasts is 10 *μ*m. We correspondingly take $\frac{1-\theta }{\theta }=6$, i.e., *θ* = 1/7. The dimensions of a lung are 12 × 31 × 41 *cm* ^3^, and there are approximately 350 million alveoli in a lung. Hence *ɛ* is extremely small.

**Table 1:** The variables of the model in units of *g*/*cm* ^3^.

*M*_1_:	density of M1 macrophages	*M*_2_:	density of M2 macrophages
*E*_0_:	density of AEC	*E*	density of activated AEC
*f*:	density of fibroblast	*m*:	density of myofibroblast
*ρ*:	density of ECM	*P*:	concentration of MCP-1
*G*:	concentration of PDGF	*T*_*β*_:	concentration of activated TGF-*β*
*Q*:	concentration of MMPs	*Q*_*r*_:	concentration of TIMP
*T*_*α*_	concentration of TNF-*α*	*I*_13_	concentration of IL-13
*S*	scar density		

We first write down all the differential equation in *T*_*ɛ*_/*A*_*ɛ*_, and then take *ɛ* → 0 to obtain the homogenized system in the cube *R*. The variables that will be used in the model are given in Table 1.

#### Equation for macrophage density

The equation for macrophage density in *T*_*ɛ*_/*A*_*ɛ*_ (coming from the blood) is given by

$$\begin{array}{ccc} \frac{\partial M_{1}}{\partial t}-D_{M}\nabla^{2}M_{1} & = & \underset{chemotaxis}{\underset{︸}{-\nabla \cdot (M_{1}\chi_{P}\nabla P)}}\underset{apoptosis}{\underset{︸}{-d_{M1}M_{1}}}+\underset{M_{2}\to M_{1}}{\underset{︸}{\lambda_{MT}\frac{T_{\alpha }}{K_{T_{\alpha }}+T_{\alpha }}M_{2}}}\underset{M_{1}\to M_{2}}{\underset{︸}{-\lambda_{M1}M_{1}}}. \end{array}$$

Macrophages are terminally differentiated cells; they do not proliferate. They differentiate from monocytes that are circulating in the blood and are attracted by MCP-1 into the lung tissue. Hence they satisfy the boundary condition

$$\begin{array}{c} D_{M}\frac{\partial M_{1}}{\partial n}+\tilde{\beta }\left(P\right)\left(M_{0}-M_{1}\right)=0\;\text{on}\;\partial T_{\varepsilon }. \end{array}$$

where $\tilde{\beta }(P)$ depends on MCP-1 concentration, *P*. Here *M*_0_ denotes the density of monoctyes in the blood, i.e., the source of *M*_1_ macrophages from the vascular system. We note that the above Robin boundary condition arises from boundary homogenization of the vascular system, as done, for example, in [34]. The term $\lambda_{MT}\frac{T_{\alpha }}{K_{T_{\alpha }}+T_{\alpha }}M_{2}$ accounts for transformation from M2 to M1 induced by TNF-*α* [21]. The term −∇⋅(*M*_1_ *χ*_*P*_∇*P*) is the chemotactic effect of MCP-1 on M1 macrophages; *χ*_*P*_ is the chemotactic coefficient. As noted in the Introduction, macrophages from blood monocytes evolve into AM [12, 16] and, in IPF, there is a shift from AM to pro-fibrotic M2 macrophages. There is also a polarization from M1 to M2 induced by MMP28 [26], and by collagen type I via CD204 receptor on M1 [17]. The term *λ*_*M*1_ *M*1 represents polarization from M1 to M2 by the above processes and possibly other processes (e.g. [35]).

We want replace the boundary condition of *M*_1_, by a spatial distribution *f*. If *D*_*M*_∇^2^ *u* = *f* in *T*_*ɛ*_/*A*_*ɛ*_, $\frac{\partial u}{\partial n}=0$ on ∂*A*_*ɛ*_, $D_{M}\frac{\partial u}{\partial n}=g$ on ∂*T*_*ɛ*_, then, by integration ∫_*T*_*ɛ*_/*A*_*ɛ*__ *fdV* = ∫_∂*T*_*ɛ*__ *gdS*. Hence

$$\begin{array}{c} \bar{f}\times \text{volume}\;\text{of}\;T_{\varepsilon }/A_{\varepsilon }=\bar{g}\times \text{area}\;\text{of}\;\partial T_{\varepsilon }, \end{array}$$

where $\bar{f}$ and $\bar{g}$ are the mean values of f and g. Since

$$\begin{array}{c} \text{volume}\;\text{of}\;T_{\varepsilon }/A_{\varepsilon }=\varepsilon^{3}\left[1-{\left(1-\theta \right)}^{3}\right]=\gamma \varepsilon^{2},\;\text{area}\;\text{of}\;\partial T_{\varepsilon }=6\varepsilon^{2}, \end{array}$$

where *γ* = 127/343, and *ɛ* is small so that $\bar{f}\approx f\;\text{and}\;\;\bar{g}\approx g$, we can replace the boundary condition of *M*1 by the spatial distribution $6ɛ\tilde{\beta }(P)/\gamma =\beta (P)$. Hence, the equation for M1 density in *T*_*ɛ*_/*A*_*ɛ*_ is given by

$$\begin{array}{ccc} \frac{\partial M_{1}}{\partial t}-D_{M}\nabla^{2}M_{1} & = & \beta \left(P\right)\left(M_{0}-M_{1}\right)-\nabla \cdot \left(M_{1}\chi_{P}\nabla P\right)-d_{M1}M_{1}\\ & & +\;\lambda_{MT}\frac{T_{\alpha }}{K_{T_{\alpha }}+T_{\alpha }}M_{2}-\lambda_{M1}M_{1}, \end{array}$$ (1)

with zero boundary flux. We take $\beta (P)=\beta \frac{P}{K_{P}+P}$, where *β* is a constant

The M2 macrophage density satisfies the equation

$$\begin{array}{ccc} \frac{\partial M_{2}}{\partial t}-D_{M}\nabla^{2}M_{2} & = & \underset{M_{1}\to M_{2}}{\underset{︸}{\lambda_{M1}M_{1}}}\underset{apoptosis}{\underset{︸}{-d_{M2}M_{2}}}\underset{M_{2}\to M_{1}}{\underset{︸}{-\lambda_{MT}\frac{T_{\alpha }}{K_{T_{\alpha }}+T_{\alpha }}M_{2}}}, \end{array}$$ (2)

where the first and last terms on the right-hand side are complimentary to the corresponding terms in Eq (1).

#### Equation for AEC density (*E*_0_ and *E*)

The equation of the inactivated AEC density is given by

$$\begin{array}{ccc} \frac{dE_{0}}{dt} & = & A_{E_{0}}(1+\underset{repair}{\underset{︸}{\frac{\lambda_{1}E_{0}I_{D}}{K_{D}+E_{0}I_{D}}}})\underset{apoptosis}{\underset{︸}{-d_{E_{0}}E_{0}(1+\delta +d_{E_{0}T}\frac{T_{\beta }}{K_{T_{\beta }}+T_{\beta }})}}\underset{E_{0}\to E}{\underset{︸}{-\lambda_{E_{0}}E_{0}I_{D}}}. \end{array}$$ (3)

In normal healthy, the production of *E*_0_ is represented by the term *A*_*E*_0__ and the death rate is represented by *d*_*E*0_ *E*_0_.

The equation for the activated AEC is

$$\begin{array}{ccc} \frac{dE}{dt} & = & \underset{activation}{\underset{︸}{\lambda_{E_{0}}E_{0}I_{D}}}\underset{EMT}{\underset{︸}{-\lambda_{EM}EI_{D}}}\underset{apoptosis}{\underset{︸}{-d_{E}E}}. \end{array}$$ (4)

In homeostasis, *I*_*D*_ = ∅, *δ* = 0 and activated TGF-*β* concentration is very small. The injury to the epithelium is expressed in two ways: (i) by activation of AEC, which is represented by term *λ*_*E*_0__ *E*_0_ *I*_*D*_, where *D* is the damaged region and *I*_*D*_ = 1 on *D* and *I*_*D*_ = 0 elsewhere, and (ii) by increased apoptosis caused by oxidative stress [4, 5] (the term *δ*) and by TGF-*β* [20, 3]. In IPF, the damaged epithelium is partially repaired by fibrocytes, and this is expressed by the term $\frac{\lambda_{1}E_{0}I_{D}}{K_{D}+E_{0}I_{D}}$ [7]. The second term of the right-hand side in Eq (4) accounts for EMT due to injury [3].

#### Equations for fibroblast density (*f*) and myofibroblast density (*m*)

The fibroblasts and myofibroblasts equations are given by:

$$\begin{array}{ccc} \frac{\partial f}{\partial t}-D_{f}\nabla^{2}f & = & \underset{source}{\underset{︸}{\lambda_{Ef}E_{0}}}+\underset{production}{\underset{︸}{\lambda_{fE}(\frac{T_{\beta }}{K_{T_{\beta }}+T_{\beta }}+\frac{I_{13}}{K_{I_{13}}+I_{13}})\frac{E}{K_{E}+E}f}}\underset{apoptosis}{\underset{︸}{-d_{f}f}}\\ & & \underset{f\to m}{\underset{︸}{-(\lambda_{mfT}\frac{T_{\beta }}{K_{T_{\beta }}+T_{\beta }}+\lambda_{mfG}\frac{G}{K_{G}+G})f}}, \end{array}$$ (5)

$$\begin{array}{ccc} \frac{\partial m}{\partial t}-D_{m}\nabla^{2}m & = & \underset{f\to m}{\underset{︸}{(\lambda_{mfT}\frac{T_{\beta }}{K_{T_{\beta }}+T_{\beta }}+\lambda_{mfG}\frac{G}{K_{G}+G})f}}\underset{apoptosis}{\underset{︸}{-d_{m}m}}. \end{array}$$ (6)

The first term on the right-hand side of Eq (5) is a source from *E*_0_-derived bFGF, which for simplicity we take to be in the form *λ*_*Ef*_ *E*_0_. As in [32], TGF-*β* and PDGF transform fibroblasts into myofibroblasts [27, 28, 29, 30]. Furthermore, TGF-*β* and IL-13 [22, 23, 24], along with E-derived bFGF, increase proliferation of fibroblasts [6, 32, 27]. For simplicity, we do not include bFGF specifically in the model, but instead represent it by *E*. The production of fibroblasts in healthy normal tissue depends on the density of AECs in homeostasis, and is represented by the term *λ*_*Ef*_ *E*_0_ [6, 20].

#### Equation for ECM density (*ρ*) and scar (*S*)

The ECM, produced by fibroblasts and myofibroblasts [27, 28, 29, 30], is degraded by MMP [36], and TGF-*β* enhances the production of ECM by myofibroblasts [27, 28, 29, 30]. The equation for the density of ECM is then given (as in [32]) by:

$$\begin{array}{ccc} \frac{\partial \rho }{\partial t} & = & \underset{production}{\underset{︸}{\lambda_{\rho f}f{\left(1-\frac{\rho }{\rho_{0}}\right)}^{+}+\lambda_{\rho m}(1+\lambda_{\rho T_{\beta }}\frac{T_{\beta }}{K_{T_{\beta }}+T_{\beta }})m}}\\ & & \;\underset{degradation}{\underset{︸}{-d_{\rho }\rho -d_{\rho Q}Q\rho }}, \end{array}$$ (7)

where ${\left(1-\frac{\rho }{\rho_{0}}\right)}^{+}=1-\frac{\rho }{\rho_{0}}$ if *ρ* < *ρ*_0_, ${\left(1-\frac{\rho }{\rho_{0}}\right)}^{+}=0$ if *ρ* ≥ *ρ*_0_.

Excessive accumulation of ECM components (particularly collagen) associated with tissue injury and inflammation, results in permanent scar formation [37]. Within each type of scar, there is considerable heterogeneity: an imbalance between MMP and TIMP activity has been implicated in the development of scar [31]. Thus a scar depends on production and deposition of ECM and disruption of normal, healthy protein cross-linking. We define the scar simply by the equation

$$\begin{array}{ccc} S & = & \lambda_{S}{\left(\rho -\rho^{*}\right)}^{+}, \end{array}$$ (8)

where ρ* is the ECM density in homeostasis and *λ*_*S*_ is a constant, but this definition is a simplified characterization of a scar since it does not account for disruption in protein cross-linking.

#### Equation for MCP-1 (*P*)

The MCP-1 equation is given by

$$\begin{array}{ccc} \frac{\partial P}{\partial t}-D_{P}\nabla^{2}P & = & \underset{production}{\underset{︸}{\lambda_{PE}E}}\underset{degradation}{\underset{︸}{-d_{P}P-d_{PM}\frac{P}{K_{P}+P}M_{1}}}, \end{array}$$ (9)

where *λ*_*PE*_ represents the growth rate by activated AEC following damage to the endothelium [32, 7, 14, 15, 1]. The last term accounts for the internalization of MCP-1 by macrophage, which may be limited due to the limited rate of receptor recycling.

#### Equations for concentrations of PDGF (*G*), MMP (*Q*), TIMP (*Q*_*r*_), TGF-*β* (*T*_*β*_), TNF-*α* (*T*_*α*_) and IL-13 (*I*_13_)

As in [32], the following sets of diffusion equations hold for *G*, *Q* and *Q*_*r*_:

$$\begin{array}{ccc} \frac{\partial G}{\partial t}-D_{G}\nabla^{2}G & = & \underset{production}{\underset{︸}{\lambda_{GM}M_{2}}}\;\underset{degradation}{\underset{︸}{-d_{G}G}}, \end{array}$$ (10)

$$\begin{array}{ccc} \frac{\partial Q}{\partial t}-D_{Q}\nabla^{2}Q & = & \underset{production}{\underset{︸}{\lambda_{QM}M_{2}}}\underset{depletion}{\underset{︸}{-d_{QQ_{r}}Q_{r}Q}}\underset{degradation}{\underset{︸}{-d_{Q}Q}}, \end{array}$$ (11)

$$\begin{array}{ccc} \frac{\partial Q_{r}}{\partial t}-D_{Q_{r}}\nabla^{2}Q_{r} & = & \underset{production}{\underset{︸}{\lambda_{Q_{r}M}M_{2}}}\underset{depletion}{\underset{︸}{-d_{Q_{r}Q}QQ_{r}}}\underset{degradation}{\underset{︸}{-d_{Q_{r}}Q_{r}}}. \end{array}$$ (12)

Note that in Eq (11), MMP is lost by binding with TIMP (second term).

As in [32], TGF-*β* is produced and activated by M2 macrophages while enhanced by IL-13 [22, 23, 24]; in addition, TGF-*β* is produced and activated by fibroblasts and AEC [12, 20]:

$$\begin{array}{ccc} \frac{\partial T_{\beta }}{\partial t}-D_{T_{\beta }}\nabla^{2}T_{\beta } & = & \underset{production}{\underset{︸}{\lambda_{T_{\beta }M}M_{2}(1+\lambda_{T_{\beta }I_{13}}\frac{I_{13}}{I_{13}+K_{I_{13}}})+\lambda_{T_{\beta }f}f\frac{E}{E+K_{E}}}}\underset{degradation}{\underset{︸}{-d_{T_{\beta }}T_{\beta }}}. \end{array}$$ (13)

TNF-*α* is produced by M1 macrophages [21], and is also produced by AEC [12, 13]:

$$\begin{array}{ccc} \frac{\partial T_{\alpha }}{\partial t}-D_{T_{\alpha }}\nabla^{2}T_{\alpha } & = & \underset{production}{\underset{︸}{\lambda_{T_{\alpha }M}M_{1}+\lambda_{T_{\alpha }E}E}}\underset{degradation}{\underset{︸}{-d_{T_{\alpha }}T_{\alpha }}}\underset{M_{2}\to M_{1}}{\underset{︸}{-\lambda_{MT_{\alpha }}\frac{T_{\alpha }}{K_{T_{\alpha }}+T_{\alpha }}M_{2}}}. \end{array}$$ (14)

IL-13 is produced by M2 macrophages [22, 23], and follows the equation

$$\begin{array}{ccc} \frac{\partial I_{13}}{\partial t}-D_{I_{13}}\nabla^{2}I_{13} & = & \underset{production}{\underset{︸}{\lambda_{I_{13}}+\lambda_{I_{13}M}M_{2}}}\underset{degradation}{\underset{︸}{-d_{I_{13}}I_{13}}}. \end{array}$$ (15)

Actually, IL-13 is also produced by TH2 cells [25]; for simplicity we do not include TH2 cells in our model but accounts for their production of IL-13 by the term *λ*_*I*_13__.

#### The homogenized equations

On the boundary of *T*_*ɛ*_/*A*_*ɛ*_ all the variables are assumed to have zero flux. Hence, each of the Eqs (1)–(14), if written in the form

$$\begin{array}{c} \frac{\partial X}{\partial t}-D_{X}\nabla^{2}X=F_{X}\left(X\right)\;\text{in}\;T_{\varepsilon }/A_{\varepsilon }, \end{array}$$ (16)

takes, after homogenization [38] (Sec. 3.1 and p.31), the following form:

$$\begin{array}{c} \gamma \frac{\partial X}{\partial t}-D_{X}\tilde{\nabla^{2}}X=\gamma F_{X}\left(X\right)\;\text{in}\;\text{the}\;\text{cube}\;R, \end{array}$$ (17)

where *γ* is the volume fraction of the tissue in each *ɛ*-cube, $\gamma =\frac{127}{343}$. Here $\tilde{\nabla^{2}}=\sum a_{ij}\frac{\partial^{2}}{\partial x_{i}\partial x_{j}}$, where the coefficient *a*_*ij*_ are computed by

$$\begin{array}{c} a_{ij}=\int_{y\in T/A}\left(\delta_{ij}+\frac{\partial \chi_{j}}{\partial x_{i}}\right)dy. \end{array}$$

where *χ*_*i*_ satisfies the equation

$$\begin{array}{c} \nabla^{2}\chi_{i}=0\;\text{in}\;T\setminus A,\;\text{with}\;\frac{\partial \chi_{i}}{\partial n}+n_{i}=0\;\text{on}\;\text{the}\;\text{boundary}\;\text{of}\;A; \end{array}$$

here $T=\frac{T_{ɛ}}{ɛ}$, $A=\frac{A_{ɛ}}{ɛ}$, *n*_*i*_ is the *i*-th component of the outward normal *n*, and *χ*_*i*_ is periodic in the directions of the three axes *x*_*j*_ (j = 1,2,3). Computing *a*_*ij*_ by finite element discretization, we find (similarly to [32]) that *a*_*ii*_ = 0.11 (i = 1,2,3) and *a*_*ij*_ = 0 if *i* ≠ *j*.

### Boundary conditions

All variables are assumed to satisfy the zero flux boundary condition on ∂*R*, the boundary of the cube *R*.

### Initial conditions

We assume initial homeostasis, that is, *λ*_*E*_0__ *E*_0_ *I*_*D*_ = 0, but with a small amount of inflammation, represented by the term *λ*_*PE*_ *E* in Eq (9). We take this term to be 10^−10^ and compute the initial values by solving the steady state equations.

In particular we find the initial values of *T*_*α*_ = 2.5 × 10^−8^, *T*_*β*_ = 2.51 × 10^−12^ and *I*_13_ = 3.2 × 10^−8^ in units of *gm*/*ml*. Taking into account that only *γ*-fraction of the space is occupied by tissue, the values $\frac{1}{\gamma }T_{\alpha }$, $\frac{1}{\gamma }I_{13}$ coincide with the concentration of *T*_*α*_ and *I*_13_ measured in the bronchial tubes of healthy lung in [39], and $\frac{1}{\gamma }T_{\beta }$ coincides with value of TGF-*β* as computed in [40].

We also compute that *E*_0_ = E* = 0.79 *g*/*cm* ^3^, *f* = f* = 4.75 × 10^−3^ *g*/*cm* ^3^, *ρ* = ρ* = 3.26 × 10^−3^ *g*/*cm* ^3^ and *I*_13_ = 1.76 × 10^−8^ *g*/*cm* ^3^ at *t* = 0.

## Results

## Numerical scheme

We briefly describe the technique used in the simulations, and for simplicity take *R* to be the unit cube, i.e., *R* = [0, 1] × [0, 1] × [0, 1]. Consider the following general diffusion equation

$$\begin{array}{c} \frac{\partial C}{\partial t}-D_{C}\nabla^{2}C=F_{C}\left(C\right), \end{array}$$

in *R* with zero flux on ∂*R*. Given three positive integers *K*_1_, *K*_2_, *K*_3_, let

$$\begin{array}{c} x_{i}=i/K_{1},\;y_{j}=j/K_{2},\;z_{k}=k/K_{3},\;0\leq i\leq K_{1},\;0\leq j\leq K_{2},\;0\leq k\leq K_{3}. \end{array}$$

Then we denote *c*_*i*, *j*, *k*_(*t*) the numerical approximation of *C*(*x*_*i*_, *y*_*j*_, *z*_*k*_, *t*), and get the following ODE system by semi-discretization:

$$\begin{array}{ccc} \frac{dc_{i,j,k}\left(t\right)}{\partial t} & = & D_{C}(K_{1}^{2}[c_{i+1,j,k}\left(t\right)+c_{i-1,j,k}\left(t\right)-2c_{i,j,k}\left(t\right)]+K_{2}^{2}[c_{i,j+1,k}\left(t\right)+c_{i,j-1,k}\left(t\right)-2c_{i,j,k}\left(t\right)]\\ & & +\;K_{3}^{2}[c_{i,j,k-1}\left(t\right)+c_{i,j,k+1}\left(t\right)-2c_{i,j,k}\left(t\right)])+F_{C}\left(c_{i,j,k}\left(t\right)\right). \end{array}$$ (18)

The Runge-kutta method is employed to solve this ODE system. The above method is used to solve the coupled system of equations of the complete model.

### Model simulation and validation

In this section, we simulate the model (1)-(17). The parameter values are listed in Tables 2 and 3 and the initial values are taken as explained above. The numerical simulation were carried out by finite difference scheme in spatial direction and Runge-Kutta method in time direction.

**Table 2:** Parameters’ description and value.

Parameter	Description	Value
*D*_*M*_	dispersion coefficient of macrophages	8.64 × 10^−7^ *cm* ^2^ day^−1^ [32]
*D*_*P*_	diffusion coefficient of MCP-1	1.728 × 10^−1^ *cm* ^2^ day^−1^ [32]
*D*_*G*_	diffusion coefficient of PDGF	8.64 × 10^−2^ *cm* ^2^ day^−1^ [32]
*D*_*Q*_	diffusion coefficient of MMP	4.32 × 10^−2^ *cm* ^2^ day^−1^ [32]
*D*_*Q*_*r*__	diffusion coefficient for TIMP	4.32 × 10^−2^ *cm* ^2^ day^−1^ [32]
*D*_*T*_*β*__	diffusion coefficient for TGF-*β*	4.32 × 10^−2^ *cm* ^2^ day^−1^ [32]
*D*_*T*_*α*__	diffusion coefficient for TNF-*α*	1.29 × 10^−2^ *cm* ^2^ day^−1^ [55]
*D*_*f*_	dispersion coefficient of fibroblasts	1.47 × 10^−6^ *cm* ^2^ day^−1^ [32]
*D*_*m*_	dispersion coefficient of myofibroblasts	1.47 × 10^−5^ *cm* ^2^ day^−1^ [32]
*D*_*I*_13__	diffusion coefficient of IL-13	1.08 × 10^−2^ *cm* ^2^ day^−1^ [40]
*D*_*T*_*α*__	diffusion coefficient for TNF-*α*	1.29 × 10^−2^ *cm* ^2^ day^−1^ [40]
*λ*_*MT*_	transition rate of M2 to M1 macrophages by TNF-*α*	5 × 10^−3^ day^−1^ [56]
*λ*_*M*1_	polarization rate of M1 to M2 macrophages	9.02 × 10^−6^ day^−1^, estimated
*λ*_*E*_0__	production rate of AEC	0.25 day^−1^ estimated
*λ*_1_	repair rate of AEC	10^−3^ *g*/*cm* ^3^ day^−1^ estimated
*λ*_*EM*_	EMT rate of AEC	1.65 × 10^−3^ day^−1^ estimated
*λ*_*T*_*β*_*M*_	production rate of TGF-*β* by macrophages	1.5 × 10^−2^ day^−1^ [32]
*λ*_*T*_*β*_*f*_	production rate of TGF-*β* by fibroblast	7.5 × 10^−3^ day^−1^ [32] & estimated
*λ*_*GM*_	production rate of PDGF by macrophages	2.4 × 10^−5^ day^−1^ [32]
*λ*_*QM*_	production rate of MMP by macrophages	3 × 10^−4^ day^−1^ [32]
*λ*_*Q*_*r*_*M*_	production rate of TIMP by macrophages	6 × 10^−5^ day^−1^ [32]
*λ*_*PE*_	activation rate of MCP-1 due to AECs	1 × 10^−8^ day^−1^ [32]
*λ*_*ρf*_	activation rate of ECM due to fibroblasts	3 × 10^−3^ day^−1^ [32]
*λ*_*ρm*_	activation rate of ECM due to myofibroblasts	6 × 10^−3^ day^−1^ [32]
*λ*_*ρT*_*β*__	activation rate of ECM due to TGF-*β*	2 [32]
*λ*_*Ef*_	activation rate of fibroblasts due to bFGF and TGF-*β*	2.5 × 10^−1^ day^−1^ [32] & estimated
*λ*_*fE*_	production rate of fibroblasts	5 × 10^−4^ day^−1^ [32] & estimated
*λ*_*mfT*_	activation rate of myofibroblasts due to TGF-*β*	0.12 day^−1^ [32]
*λ*_*mfG*_	activation rate of myofibroblasts due to PDGF	0.12 day^−1^ [32]
*λ*_*T*_*α*_*M*_	activation rate of TNF-*α* due tomacrophage	1.39 × 10^−5^ day^−1^ [57]
*λ*_*T*_*α*_*E*_	activation rate of TNF-*α* due tomacrophage	6.9 × 10^−6^ day^−1^ [57] & estimated
*λ*_*I*_13__	production rate of IL-13 by Th2 cells	2.12 × 10^−7^ g/ml day^−1^ [40]
*λ*_*I*_13_*M*_	production rate of IL-13 by macrophages	3.98 × 10^−4^ day^−1^ [40]
*d*_*M*_2__	death rate of macrophages	0.015 day^−1^ [32]
*d*_*M*_1__	death rate of macrophages	0.02 day^−1^ [56, 58]
*d*_*E*_	death rate of AECs	1.65 × 10^−2^ day^−1^ [32]
*d*_*E*_0__	death rate of AECs	1.65 × 10^−2^ day^−1^ [32]
*d*_*E*_0_*T*_	death rate of AECs	1.65 × 10^−3^ day^−1^ [32]
*δ*	increased death rate of AECs by injury	1 × 10^−3^ day^−1^, estimated
*d*_*ρ*_	degradation rate of ECM	0.37 day^−1^ [32]
*d*_*P*_	degradation rate of MCP-1	1.73 day^−1^ [32]
*d*_*PM*_	internalization rate of MCP-1 by M1 macrophages	2.08 × 10^−4^ day^−1^ [32]
*d*_*G*_	degradation rate of PDGF	3.84 day^−1^ [32]
*d*_*QQ*_*r*__	binding rate of MMP to TIMP	4.98 × 10^8^ *cm* ^3^ *g* ^−1^ day^−1^ [32]
*d*_*Q*_*r*_*Q*_	binding rate of TIMP to MMP	1.04 × 10^9^ *cm* ^3^ *g* ^−1^ day^−1^ [32]
*d*_*Q*_	degradation rate of MMP	4.32 day^−1^ [32]
*d*_*Q*_*r*__	degradation rate of TIMP	21.6 day^−1^ [32]
*d*_*ρQ*_	degradation rate of ECM due to MMP	2.59 × 10^7^ *cm* ^3^ *g* ^−1^ day^−1^ [32]
*d*_*T*_*β*__	degradation rate of TGF-*β*	3.33 × 10^2^ day^−1^ [32]
*d*_*f*_	death rate of fibroblasts	1.66 × 10^−2^ day^−1^ [32]
*d*_*m*_	death rate of myofibroblasts	1.66 × 10^−2^ day^−1^ [32]
*d*_*T*_*α*__	degradation rate of TNF-*α*	55.45 day^−1^ [59]
*d*_*I*_13__	degradation rate of IL-13	12.47 day^−1^ [40]

**Table 3:** Parameters’ description and value.

Parameter	Description	Value
*χ*_*P*_	chemotactic sensitivity parameter by MCP-1	10 *cm* ^5^ *g* ^−1^ day^−1^ [32]
*A*_*E*0_	intrinsic AEC proliferation	8.27 × 10^−3^ *g*/*cm* ^3^ day^−1^ [32]
*K*_*G*_	PDGF saturation for activation of myofibroblasts	1.5 × 10^−8^ *gcm* ^−3^ [32]
*K*_*T*_*β*__	TGF-*β* saturation for apoptosis of AECs	1 × 10^−10^ *gcm* ^−3^ [32]
*K*_*P*_	MCP-1 saturation for influx of macrophages	5 × 10^−9^ *gcm* ^−3^ [32]
*K*_*T*_*α*__	TNF-*α* saturation	5 × 10^−7^ *gcm* ^−3^ [40]
*K*_*I*_13__	IL-13 saturation	2 × 10^−7^ g/*cm* ^3^ [40]
*K*_*E*_	AEC saturation	0.1 g/*cm* ^3^, estimated
*ρ*_0_	ECM saturation	10^−2^ *gcm* ^−3^ [32]
ρ*	ECM density in health	3.26 × 10^−3^ *gcm* ^−3^ estimated
E*	TEC density in health	0.799 *gcm* ^−3^ estimated
f*	fibroblast density in health	4.75 × 10^−3^ *gcm* ^−3^ estimated
*M*_0_	source/influx of macrophages from blood	5 × 10^−5^ *gcm* ^−3^ [32]
*β*	influx rate of macrophages into interstitium	0.2 *cm* ^−1^ [32]

**Fig 3:**
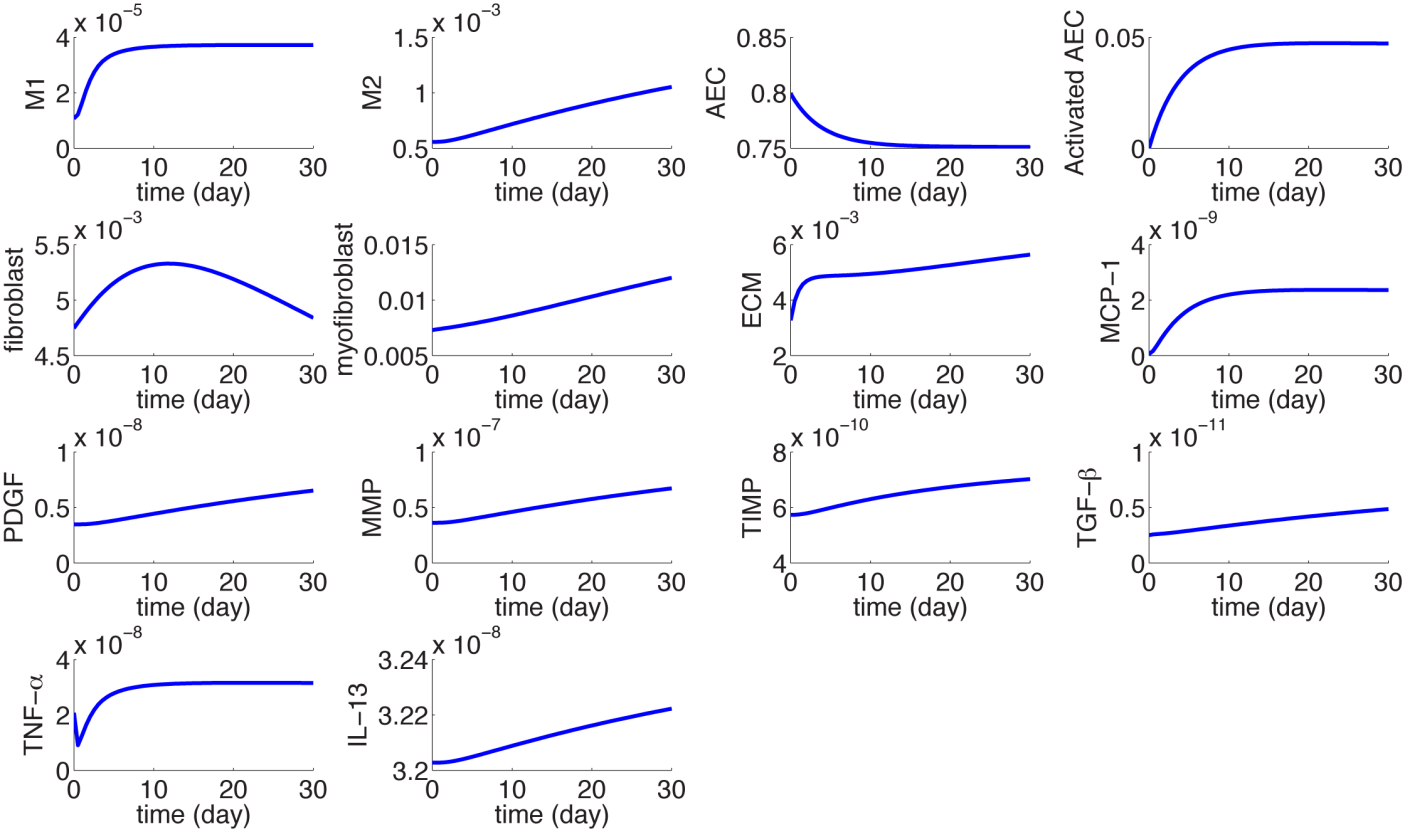
The dynamics of the average concentrations of cells and cytokines in units of *gm*/*cm* ^3^ from homeostasis at day 0 to day 30. *I*_*D*_ = 0.3 × 0.3 × 0.3 *cm* ^3^.

Fig 3 shows the dynamics of the average densities of cells and concentrations of cytokines for 30 days.

**Fig 4:**
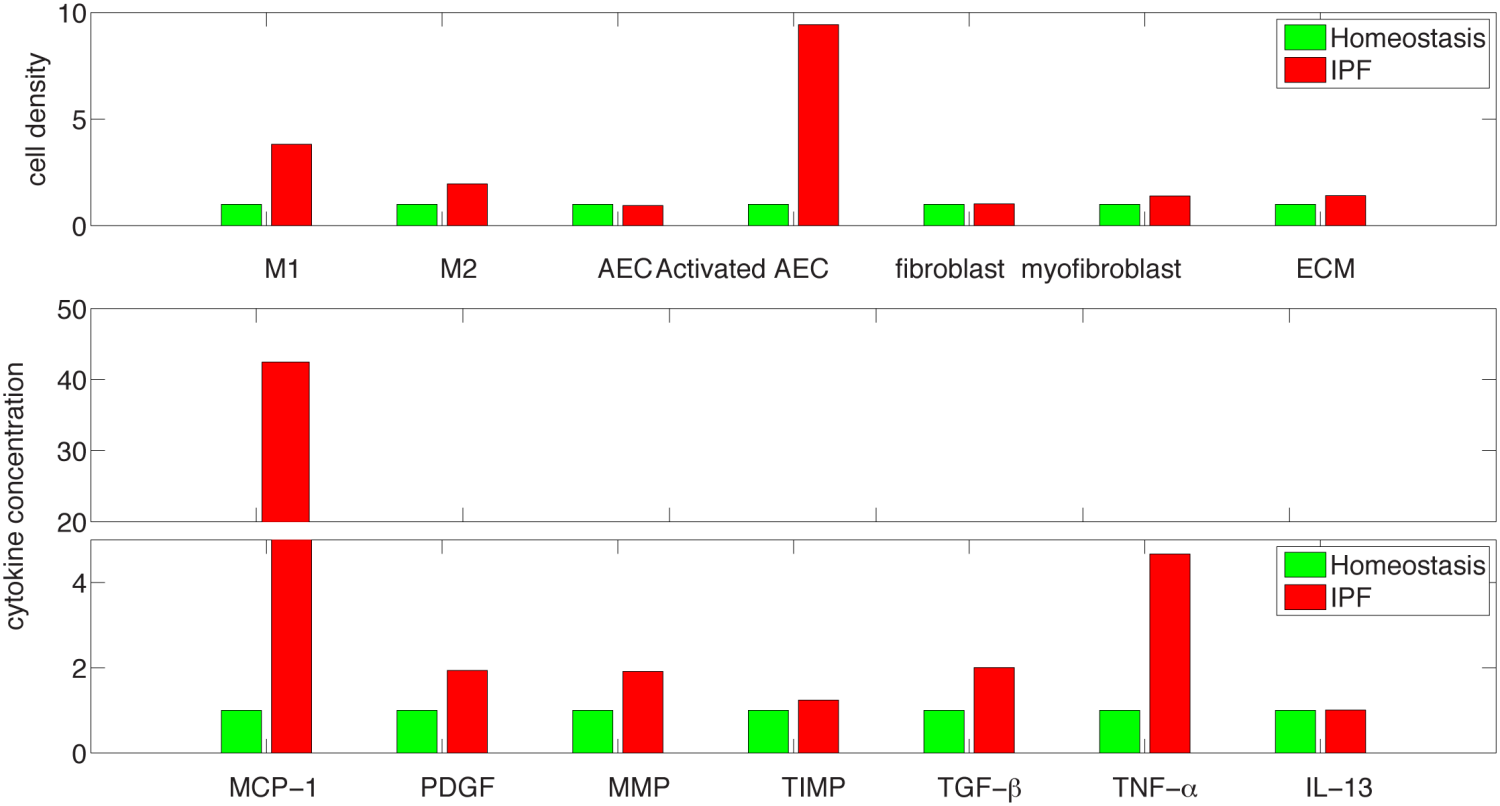
Comparison of cells and cytokines for IPF and healthy control at day 30 from the beginning of the disease (in fraction of healthy control).

Fig 4 shows histogram of cells and cytokines in disease vs. homeostasis. The simulation results for MMP and TIMP shown in Fig 4 are in agreement with the experimental results, reported in [41] for protein concentration human lung tissue with IPF (n = 16 human subjects) and control (n = 6 human subjects). Indeed, although (in [41]) MMP 7 (for IPF) is nearly 4 times the level of MMP7 for control, all other MMPs (1,2,9,13) increased approximately twice or just a little more than twice, while the relatively small concentration of MMP8 decreased to 25% of the control level. The simulation results for TIMP shows an increase of 20% in the protein concentration for IPF vs. control, which is the same as in human lung tissues reported in [41] for TIMP-1,2,3. Levels of mRNA expression relate to levels of the translated proteins. The mRNA of TGF-*β* reported in [42] (which can also be deduced from [43]) shows increase by at least twice in IPF vs. control; this increase is the same for the TGF-*β* protein shown in Fig 4. However, we cannot make too much out of this comparison since TGF-*β* has to be activated post transcriptionally to be biologically active [20]. The mRNA expressions of TNF-*α* and PDGF reported in [43] show increased levels in IPF patients, which is in qualitative agreement with the increase in protein levels shown in Fig 4.

**Fig 5:**
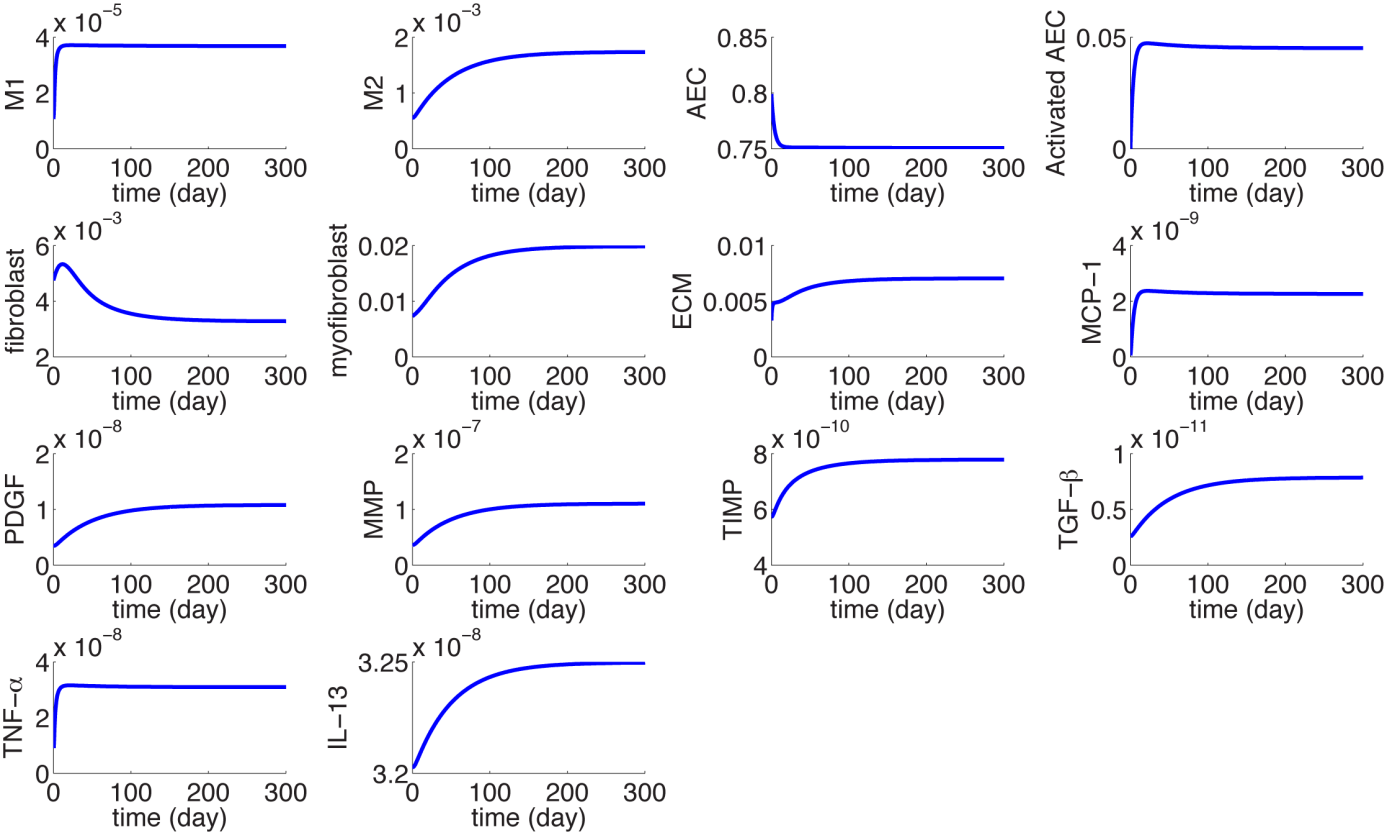
The dynamics of the average concentrations of cells and cytokines in units of *gm*/*cm* ^3^ from homeostasis at day 0 to day 300. *I*_*D*_ = 0.3 × 0.3 × 0.3 *cm* ^3^.

**Fig 6:**
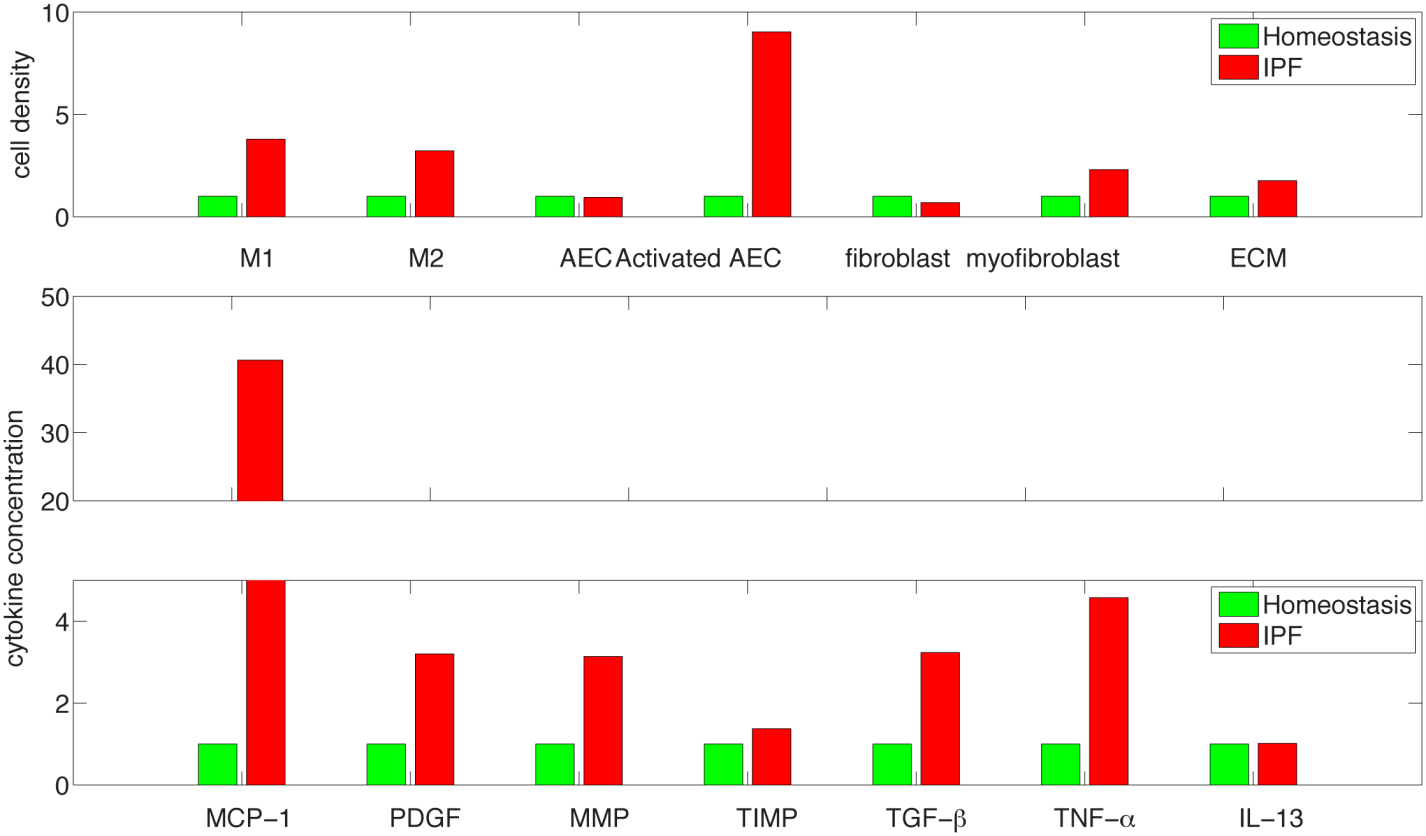
Comparison of cells and cytokines for IPF and healthy control at day 300 from the beginning of the disease (in fraction of healthy control).

Figs 5 and 6 are simulations of the disease for a larger period of 300 days. We see that the disease continue to grow but at slower rate.

### Treatment studies

**Fig 7:**
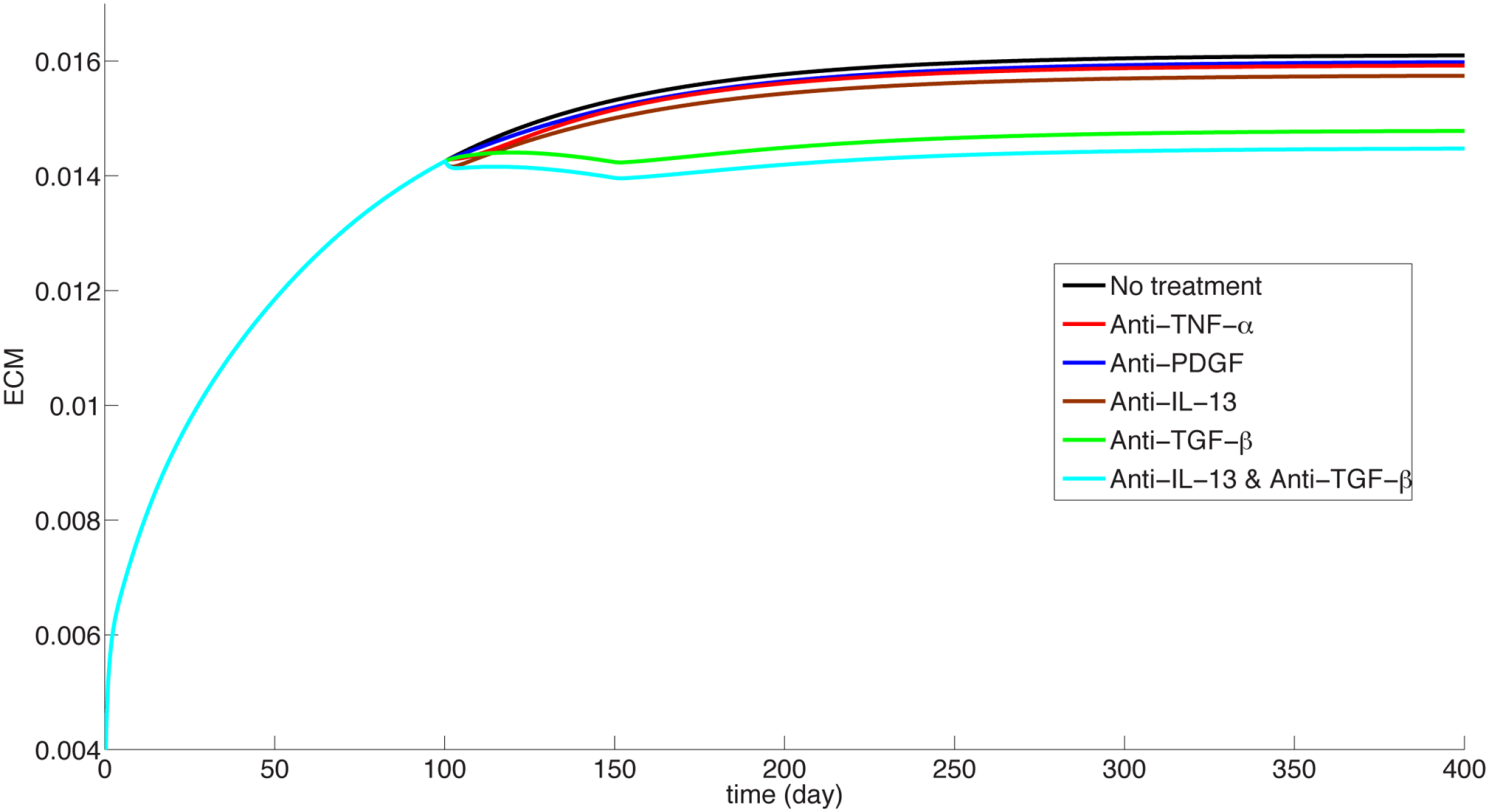
Treatment studies for the mild case. ECM is in units of *gm*/*cm* ^3^.

**Fig 8:**
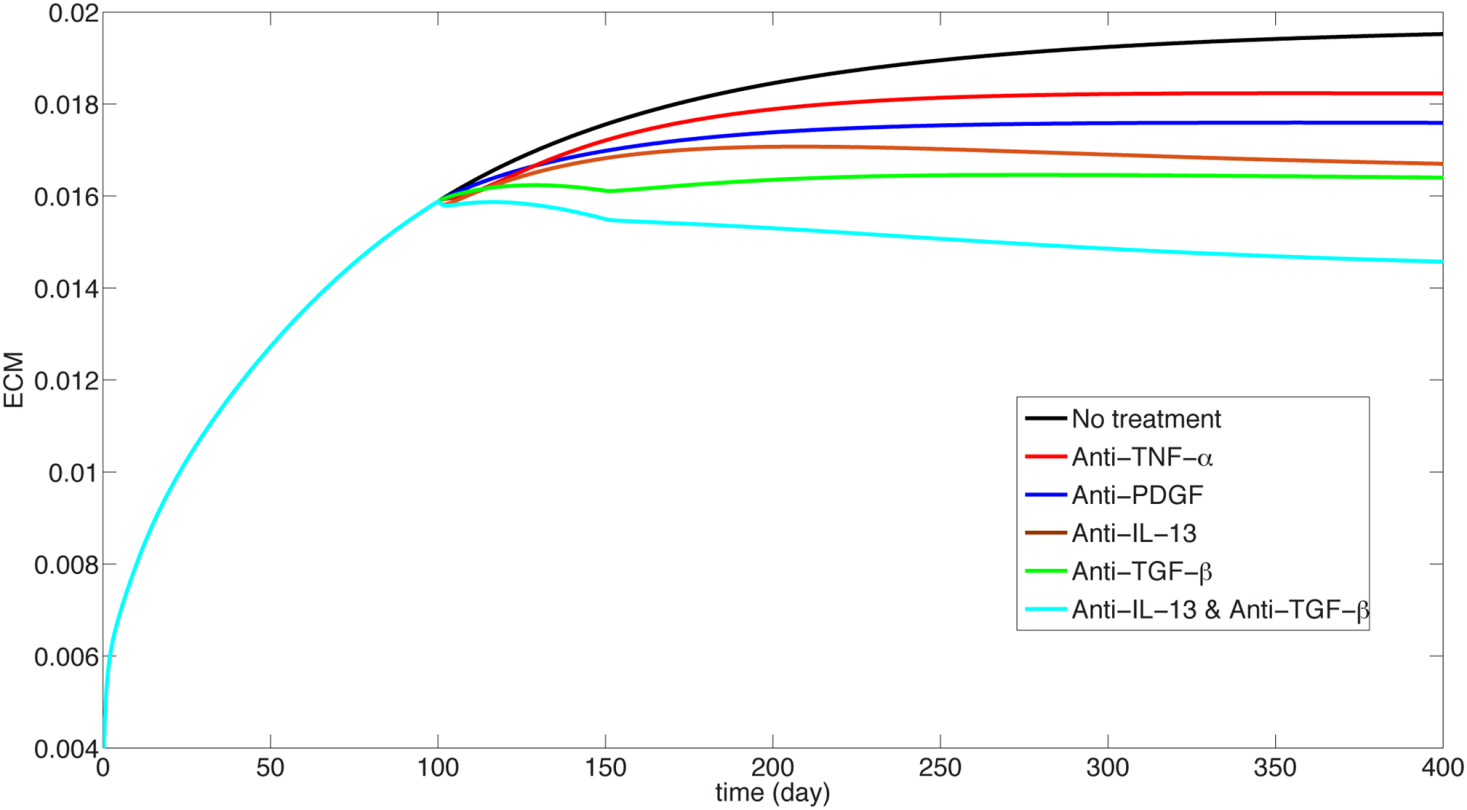
Treatment studies for the severe case. ECM is in units of *gm*/*cm* ^3^.

We can use the model to explore potential drugs. Such drugs could be, for instance, anti-TGF-*β*, anti-PDGF, anti-IL-13 or anti-TNF-*α*. Fig 7 displays the effect of treatment for mild case of IPF, namely *I*_*D*_ = 0.3 × 0.3 and *λ*_*E*_0__ = 2.5 × 10^−3^ day^−1^, and Fig 8 displays the effect of treatment for severe case of IPF, namely, *I*_*D*_ = 0.5 × 0.5 and *λ*_*E*_0__ = 3 × 10^−3^ day^−1^

#### Anti TNF-*α*

To implement the effect of anti-TNF-*α* (TNF-*α* receptor that inactivates TNF-*α* and thus blocks TNF-*α* activity [44]), we need to modify the model replacing *λ*_*MT*_ in Eqs (1) (2) by *λ*_*MT*_/(1 + *B*_1_) to represent the inhibition of the activity of TNF-*α*. We assume that the drug is administered starting at day 100 from the beginning of the disease. The red curve in Figs 7 and 8 show the effect of the drug on the ECM average concentration (with *B*_1_ = 1) over a period of 300 days. The corresponding scar has a similar curve and hence it is not given here. We see that the drug has no effect on reducing the ECM. This is in agreement with clinical phase 2 trials with Etanercept reported in [44].

The effect of the drug is introduced gradually over a period of 20 days, that is, we actually take *θ*(*t*)*B*_1_ instead of *B*_1_, where *θ*(*t*) increases linearly from 0 to 1 over a period of 20 days. The same procedure is used in treatment of the subsequent drugs.

#### Anti-PDGF

We next consider anti-PDGF treatment, by Imatinib, an inhibitor of PDGFR and thus a blocker of PDGF activity [45]. In our model this corresponds to replacing, in Eqs (5) and (6), *λ*_*mfG*_ by *λ*_*mfG*_/(1 + *B*_2_). The green curve in Figs 7 and 8 show the effect of the drug on ECM for *B*_2_ = 1. We see that the drug does not confer significant benefit, which is in agreement with phase 2 study with Imatinib.

#### Anti-IL-13

We next consider anti-IL-13, monoclonal antibody, a drug currently in early phase clinical trials. Tralokinamab and lebrikizumab are two drugs delivering antibody that blocks the action of IL-13. To implement their effect in our model we need to replace *λ*_*T*_*β*_*I*_13__ in Eq (13) by *λ*_*T*_*β*_*I*_13__/(1 + *B*_3_). With the choice of *B*_3_ = 1, the blue curve in Figs 7 and 8 show no significant benefits; this seems to suggest that a moderate level of dosing will not be effective.

#### Anti-TGF-*β*

We finally consider an anti-TGF*β* drug, such as Pirfenidone [46] which was recently approved in the United States. In our model we need to replace *λ*_*T*_*β*_*M*_ and *λ*_*T*_*β*_*f*_ by *λ*_*T*_*β*_*M*_/(1 + *A*) and *λ*_*T*_*β*_*f*_/(1 + *A*), and *T*_*β*_ by *T*_*β*_/(1 + *B*) in all terms where *T*_*β*_ acts to promote fibrosis. In the previous examples we showed that the drug has no benfits even at the level *B* = 1. For the present anti-TGF-*β* drug we demonstrate a clear benefit already with small A and B. Indeed, the cyan curve in Figs 7 and 8 show the effect of the drug on ECM for *A* = *B* = 0.1. We see that in terms of ECM, the drug could be effective in stopping, or even slowly decreasing fibrosis.

## Discussion

IPF is a disease which exhibits, as in cutaneous wounds, both pro-inflammatory features when the alveolar epithelium is damaged and AECs begin to secrete pro-inflammatory mediators, and anti-inflammatory features associated with unsuccessful repair processes.

In this paper we developed for the first time a mathematical model for IPF. The model includes many of the principal players of cells and cytokines associated with the disease. The complex geometry of the lung alveoli is simplified by using the averaging method of homogenization, which provides a way to calculate the effective interactions among the cells and cytokines. The simulations of the model agree with lung tissue data that are available from human patients. The model can be used to explore the effect of drug treatment. Indeed, we used the model to explore the treatment of IPF by anti-TNF-*α*, anti-PDGF, anti-IL-13 and anti-TGF-*β*. We found that the first three drugs did not confer any benefits, while the last drug, pirfenidone, could be effective in stopping, or even slowly decreasing fibrosis.

We can use the model to explore novel therapeutic approaches to the treatment of IPF. For example, what will be the effect of combining two anti-fibrotic drugs? From Figs 5 and 7 we see that anti-TGF-*β* is the most effective drug to slow the IPF progression (with *A* = *B* = 0.1) and anti-IL-13 has only very mild benefits (with *B*_3_ = 1). However if we combine these two drugs (at the same respective levels) we obtain significant improvement of over anti-TGF-*β* alone, especially in the case of severe case of IPF, as seen in the bottom curves in Figs 5 and 7. We propose this result as an hypothesis that could be checked in clinical trials.

The present model should be viewed as a first step in the development a more comprehensive study of IPF. Such a study should include altered DNA methylation [47, 48], epigenetic and environmental factors [49], gene mutation (e.g. of surfactant protein [50]), polymorphism (e.g. of IL-10 [51], IL-4 [52], Muc5B [53]), and telomerase mutations [54].

## References

[1] Selman M, Pardo A. Revealing the pathogenic and aging-related mechanisms of the enigmatic idiopathic pulmonary fibrosis. an integral model. Am J Respir Crit Care Med. 2014 5;189(10):1161–1172. 10.1164/rccm.201312-2221PP 24641682

[2] Bargagli E, Prasse A, Olivieri C, Muller-Quernheim J, Rottoli P. Macrophage-derived biomarkers of idiopathic pulmonary fibrosis. Pulm Med. 2011;2011:717130 10.1155/2011/717130 21637368 PMC3101790

[3] W NP. Epithelial fibroblast triggering and interactions in pulmonary fibrosis. Eur Respir Rev. 2008 5;17(109):123–129. 10.1183/09059180.00010904

[4] Daniil ZD, Papageorgiou E, Koutsokera A, Kostikas K, Kiropoulos T, Papaioannou AI, et al Serum levels of oxidative stress as a marker of disease severity in idiopathic pulmonary fibrosis. Pulm Pharmacol Ther. 2008;21(1):26–31. 10.1016/j.pupt.2006.10.005 17161968

[5] Kliment CR, Oury TD. Oxidative stress, extracellular matrix targets, and idiopathic pulmonary fibrosis. Free Radic Biol Med. 2010 9;49(5):707–717. 10.1016/j.freeradbiomed.2010.04.036 20452419 PMC13063126

[6] Camelo A, Dunmore R, Sleeman MA, Clarke DL. The epithelium in idiopathic pulmonary fibrosis: breaking the barrier. Front Pharmacol. 2014 1;4:173 10.3389/fphar.2013.00173 24454287 PMC3887273

[7] Herzog EL, Bucala R. Fibrocytes in health and disease. Exp Hematol. 2010 7;38(7):548–556. 10.1016/j.exphem.2010.03.004 20303382 PMC3136351

[8] Malli F, Koutsokera A, Paraskeva E, Zakynthinos E, Papagianni M, Makris D, et al Endothelial progenitor cells in the pathogenesis of idiopathic pulmonary fibrosis: an evolving concept. PLoS ONE. 2013;8(1):e53658 10.1371/journal.pone.0053658 23341966 PMC3544914

[9] Bringardner BD, Baran CP, Eubank TD, Marsh CB. The role of inflammation in the pathogenesis of idiopathic pulmonary fibrosis. Antioxid Redox Signal. 2008 2;10(2):287–301. 10.1089/ars.2007.1897 17961066 PMC2737712

[10] Homer RJ, Elias JA, Lee CG, Herzog E. Modern concepts on the role of inflammation in pulmonary fibrosis. Arch Pathol Lab Med. 2011 6;135(6):780–788. 21631273 10.5858/2010-0296-RA.1

[11] Zoz DF, Lawson WE, Blackwell TS. Idiopathic pulmonary fibrosis: a disorder of epithelial cell dysfunction. Am J Med Sci. 2011 6;341(6):435–438. 10.1097/MAJ.0b013e31821a9d8e 21613930 PMC3103044

[12] Boorsma CE, Draijer C, Melgert BN. Macrophage heterogeneity in respiratory diseases. Mediators Inflamm. 2013;2013:769214 10.1155/2013/769214 23533311 PMC3600198

[13] McRitchie DI, Isowa N, Edelson JD, Xavier AM, Cai L, et al Production of tumour necrosis factor alpha by primary cultured rat alveolar epithelial cells. Cytokine. 2000 6;12(6):644–654. 10.1006/cyto.1999.0656 10843740

[14] Iyonaga K, Takeya M, Saita N, Sakamoto O, Yoshimura T, Ando M, et al Monocyte chemoattractant protein-1 in idiopathic pulmonary fibrosis and other interstitial lung diseases. Hum Pathol. 1994 5;25(5):455–463. 10.1016/0046-8177(94)90117-1 8200639

[15] Selman M, Pardo A. Role of epithelial cells in idiopathic pulmonary fibrosis: from innocent targets to serial killers. Proc Am Thorac Soc. 2006 6;3(4):364–372. 10.1513/pats.200601-003TK 16738202

[16] Landsman L, Jung S. Lung macrophages serve as obligatory intermediate between blood monocytes and alveolar macrophages. J Immunol. 2007 9;179(6):3488–3494. 10.4049/jimmunol.179.6.3488 17785782

[17] Stahl M, Schupp J, Jager B, Schmid M, Zissel G, Muller-Quernheim J, et al Lung collagens perpetuate pulmonary fibrosis via CD204 and M2 macrophage activation. PLoS ONE. 2013;8(11):e81382 10.1371/journal.pone.0081382 24278429 PMC3835428

[18] Pechkovsky DV, Prasse A, Kollert F, Engel KM, Dentler J, Luttmann W, et al Alternatively activated alveolar macrophages in pulmonary fibrosis-mediator production and intracellular signal transduction. Clin Immunol. 2010 10;137(1):89–101. 10.1016/j.clim.2010.06.017 20674506

[19] Kuwano K, Hagimoto N, Nakanishi Y. The role of apoptosis in pulmonary fibrosis. Histol Histopathol. 2004 7;19(3):867–881. 15168350 10.14670/HH-19.867

[20] Sakai N, Tager AM. Fibrosis of two: Epithelial cell-fibroblast interactions in pulmonary fibrosis. Biochim Biophys Acta. 2013 7;1832(7):911–921. 10.1016/j.bbadis.2013.03.001 23499992 PMC4041487

[21] Redente EF, Keith RC, Janssen W, Henson PM, Ortiz LA, Downey GP, et al Tumor necrosis factor-alpha accelerates the resolution of established pulmonary fibrosis in mice by targeting profibrotic lung macrophages. Am J Respir Cell Mol Biol. 2014 4;50(4):825–837. 10.1165/rcmb.2013-0386OC 24325577 PMC4068926

[22] Clarke DL, Carruthers AM, Mustelin T, Murray LA. Matrix regulation of idiopathic pulmonary fibrosis: the role of enzymes. Fibrogenesis Tissue Repair. 2013;6(1):20 10.1186/1755-1536-6-20 24279676 PMC4176485

[23] Fichtner-Feigl S, Strober W, Kawakami K, Puri RK, Kitani A. IL-13 signaling through the IL-13alpha2 receptor is involved in induction of TGF-beta1 production and fibrosis. Nat Med. 2006 1;12(1):99–106. 10.1038/nm1332 16327802

[24] Jakubzick C, Choi ES, Joshi BH, Keane MP, Kunkel SL, Puri RK, et al Therapeutic attenuation of pulmonary fibrosis via targeting of IL-4- and IL-13-responsive cells. J Immunol. 2003 9;171(5):2684–2693. 10.4049/jimmunol.171.5.2684 12928422

[25] Vasakova M, Striz I, Slavcev A, Jandova S, Kolesar L, Sulc J. Th1/Th2 cytokine gene polymorphisms in patients with idiopathic pulmonary fibrosis. Tissue Antigens. 2006 3;67(3):229–232. 10.1111/j.1399-0039.2006.00560.x 16573560

[26] Gharib SA, Johnston LK, Huizar I, Birkland TP, Hanson J, Wang Y, et al MMP28 promotes macrophage polarization toward M2 cells and augments pulmonary fibrosis. J Leukoc Biol. 2014 1;95(1):9–18. 10.1189/jlb.1112587 23964118 PMC3868192

[27] Lomas NJ, Watts KL, Akram KM, Forsyth NR, Spiteri MA. Idiopathic pulmonary fibrosis: immunohistochemical analysis provides fresh insights into lung tissue remodelling with implications for novel prognostic markers. Int J Clin Exp Pathol. 2012;5(1):58–71. 22295148 PMC3267487

[28] Murray LA, Chen Q, Kramer MS, Hesson DP, Argentieri RL, Peng X, et al TGF-beta driven lung fibrosis is macrophage dependent and blocked by Serum amyloid P. Int J Biochem Cell Biol. 2011 1;43(1):154–162. 10.1016/j.biocel.2010.10.013 21044893

[29] Tatler AL, Jenkins G. TGF-beta activation and lung fibrosis. Proc Am Thorac Soc. 2012 7;9(3):130–136. 10.1513/pats.201201-003AW 22802287

[30] Xiao L, Du Y, Shen Y, He Y, Zhao H, Li Z. TGF-beta 1 induced fibroblast proliferation is mediated by the FGF-2/ERK pathway. Front Biosci (Landmark Ed). 2012;17:2667–2674. 10.2741/4077 22652804

[31] Ulrich D, Ulrich F, Unglaub F, Piatkowski A, Pallua N. Matrix metalloproteinases and tissue inhibitors of metalloproteinases in patients with different types of scars and keloids. J Plast Reconstr Aesthet Surg. 2010 6;63(6):1015–1021. 10.1016/j.bjps.2009.04.021 19464975

[32] Hao W, Rovin BH, Friedman A. Mathematical model of renal interstitial fibrosis. Proc Natl Acad Sci USA. 2014 9;111(39):14193–14198. 10.1073/pnas.1413970111 25225370 PMC4191765

[33] Ochs M, Nyengaard JR, Jung A, Knudsen L, Voigt M, Wahlers T, et al The number of alveoli in the human lung. Am J Respir Crit Care Med. 2004 1;169(1):120–124. 10.1164/rccm.200308-1107OC 14512270

[34] Xue C, Friedman A, Sen CK. A mathematical model of ischemic cutaneous wounds. Proc Natl Acad Sci USA. 2009 9;106(39):16782–16787. 10.1073/pnas.0909115106 19805373 PMC2757812

[35] Park SW, Ahn MH, Jang HK, Jang AS, Kim DJ, Koh ES, et al Interleukin-13 and its receptors in idiopathic interstitial pneumonia: clinical implications for lung function. J Korean Med Sci. 2009 8;24(4):614–620. 10.3346/jkms.2009.24.4.614 19654941 PMC2719183

[36] Orbe J, Rodriguez JA, Arias R, Belzunce M, Nespereira B, Perez-Ilzarbe M, et al Antioxidant vitamins increase the collagen content and reduce MMP-1 in a porcine model of atherosclerosis: implications for plaque stabilization. Atherosclerosis. 2003 3;167(1):45–53. 10.1016/S0021-9150(02)00392-1 12618267

[37] Mann CJ, Perdiguero E, Kharraz Y, Aguilar S, Pessina P, Serrano AL, et al Aberrant repair and fibrosis development in skeletal muscle. Skelet Muscle. 2011;1(1):21 10.1186/2044-5040-1-21 21798099 PMC3156644

[38] Jikov VV, Kozlov SM, Oleinik OA. Homogenization of differential operators and integral functionals. Springer-Verlag 1994;.

[39] Crouser ED, Culver DA, Knox KS, Julian MW, Shao G, Abraham S, et al Gene expression profiling identifies MMP-12 and ADAMDEC1 as potential pathogenic mediators of pulmonary sarcoidosis. Am J Respir Crit Care Med. 2009 5;179(10):929–938. 10.1164/rccm.200803-490OC 19218196 PMC2684019

[40] Hao W, Crouser ED, Friedman A. Mathematical model of sarcoidosis. Proc Natl Acad Sci USA. 2014 11;111(45):16065–16070. 10.1073/pnas.1417789111 25349384 PMC4234561

[41] Nkyimbeng T, Ruppert C, Shiomi T, Dahal B, Lang G, Seeger W, et al Pivotal role of matrix metalloproteinase 13 in extracellular matrix turnover in idiopathic pulmonary fibrosis. PLoS ONE. 2013;8(9):e73279 10.1371/journal.pone.0073279 24023851 PMC3759404

[42] Bergeron A, Soler P, Kambouchner M, Loiseau P, Milleron B, Valeyre D, et al Cytokine profiles in idiopathic pulmonary fibrosis suggest an important role for TGF-beta and IL-10. Eur Respir J. 2003 7;22(1):69–76. 10.1183/09031936.03.00014703 12882453

[43] Cho JH, Gelinas R, Wang K, Etheridge A, Piper MG, Batte K, et al Systems biology of interstitial lung diseases: integration of mRNA and microRNA expression changes. BMC Med Genomics. 2011;4:8 10.1186/1755-8794-4-8 21241464 PMC3035594

[44] Raghu G, Brown KK, Costabel U, Cottin V, du Bois RM, Lasky JA, et al Treatment of idiopathic pulmonary fibrosis with etanercept: an exploratory, placebo-controlled trial. Am J Respir Crit Care Med. 2008 11;178(9):948–955. 10.1164/rccm.200709-1446OC 18669816

[45] Daniels CE, Lasky JA, Limper AH, Mieras K, Gabor E, Schroeder DR, et al Imatinib treatment for idiopathic pulmonary fibrosis: Randomized placebo-controlled trial results. Am J Respir Crit Care Med. 2010 3;181(6):604–610. 10.1164/rccm.200906-0964OC 20007927

[46] Takeda Y, Tsujino K, Kijima T, Kumanogoh A. Efficacy and safety of pirfenidone for idiopathic pulmonary fibrosis. Patient Prefer Adherence. 2014;8:361–370. 10.2147/PPA.S37233 24711695 PMC3968083

[47] Sanders YY, Ambalavanan N, Halloran B, Zhang X, Liu H, et al Altered DNA methylation profile in idiopathic pulmonary fibrosis. Am J Respir Crit Care Med. 2012 9;186(6):525–535. 10.1164/rccm.201201-0077OC 22700861 PMC3480526

[48] Rabinovich EI, Kapetanaki MG, Steinfeld I, Gibson KF, Pandit KV, Yu G, et al Global methylation patterns in idiopathic pulmonary fibrosis. PLoS ONE. 2012;7(4):e33770 10.1371/journal.pone.0033770 22506007 PMC3323629

[49] Yang IV, Schwartz DA. Epigenetics of idiopathic pulmonary fibrosis. Transl Res. 2015 1;165(1):48–60. 10.1016/j.trsl.2014.03.011 24746870 PMC4182166

[50] Nogee LM, Dunbar AE, Wert S, Askin F, Hamvas A, Whitsett JA. Mutations in the surfactant protein C gene associated with interstitial lung disease. Chest. 2002 3;121(3 Suppl):20S–21S. 10.1378/chest.121.3_suppl.20S 11893657

[51] Whittington HA, Freeburn RW, Godinho SI, Egan J, Haider Y, Millar AB. Analysis of an IL-10 polymorphism in idiopathic pulmonary fibrosis. Genes Immun. 2003 6;4(4):258–264. 10.1038/sj.gene.6363959 12761561

[52] Vasakova M, Sterclova M, Matej R, Olejar T, Kolesar L, Skibova J, et al IL-4 polymorphisms, HRCT score and lung tissue markers in idiopathic pulmonary fibrosis. Hum Immunol. 2013 10;74(10):1346–1351. 10.1016/j.humimm.2013.07.011 23911740

[53] Seibold MA, Wise AL, Speer MC, Steele MP, Brown KK, et al A common MUC5B promoter polymorphism and pulmonary fibrosis. N Engl J Med. 2011 4;364(16):1503–1512. 10.1056/NEJMoa1013660 21506741 PMC3379886

[54] Armanios M. Telomerase mutations and the pulmonary fibrosis-bone marrow failure syndrome complex. N Engl J Med. 2012 7;367(4):384; author reply 384. 10.1056/NEJMc1206730 22830481

[55] Hao W, Friedman A. The LDL-HDL Profile Determines the Risk of Atherosclerosis: A Mathematical Model. PLoS ONE. 2014;9(3):e90497 10.1371/journal.pone.0090497 24621857 PMC3951264

[56] Day J, Friedman A, Schlesinger LS. Modeling the immune rheostat of macrophages in the lung in response to infection. Proc Natl Acad Sci USA. 2009 7;106(27):11246–11251. 10.1073/pnas.0904846106 19549875 PMC2708732

[57] Alleva DG, Burger CJ, Elgert KD. Tumor-induced regulation of suppressor macrophage nitric oxide and TNF-alpha production. Role of tumor-derived IL-10, TGF-beta, and prostaglandin E2. J Immunol. 1994 8;153(4):1674–1686. 8046239

[58] Murphy J, Summer R, Wilson AA, Kotton DN, Fine A. The prolonged life-span of alveolar macrophages. Am J Respir Cell Mol Biol. 2008 4;38(4):380–385. 10.1165/rcmb.2007-0224RC 18192503 PMC2274942

[59] Oliver JC, Bland LA, Oettinger CW, Arduino MJ, McAllister SK, Aguero SM, et al Cytokine kinetics in an in vitro whole blood model following an endotoxin challenge. Lymphokine Cytokine Res. 1993 4;12(2):115–120. 8324076

